# Spongiotic Pattern in Pemphigus: A Retrospective Observational Single-Center Study

**DOI:** 10.3390/dermatopathology9020022

**Published:** 2022-05-20

**Authors:** Ivan Arni C. Preclaro, Yu-Hung Wu

**Affiliations:** 1 Department of Dermatology, MacKay Memorial Hospital, Taipei 10449, Taiwan; ivanpreclaro@gmail.com; 2 Department of Medicine, MacKay Medical College, New Taipei City 25245, Taiwan

**Keywords:** spongiosis, pemphigus, eczema, Langerhans cell microabscess

## Abstract

Pemphigus is a chronic blistering disorder caused by autoantibodies that target desmosomal proteins in the epidermis. Acantholysis may be absent, and pemphigus may present only with spongiosis and vesiculation, thereby leading to a misdiagnosis of eczema. Herein, we conducted a retrospective, observational, single-center study to establish a pattern of spongiosis in cases of pemphigus confirmed by direct immunofluorescence. Immunopathologically diagnosed pemphigus specimens from 2001 to 2020 were retrieved, and specimens with spongiosis were analyzed for the following features: vesiculation, acantholysis, spongiosis, inflammatory cells in the epidermis, and inflammation in the dermis. Cases of spongiotic dermatitis were used as control. Out of 99 immunopathologically diagnosed pemphigus specimens, 41 samples with spongiosis were identified. About one quarter of the specimens did not have acantholysis. Spongiosis in the middle to lower thirds of the perilesional epidermis (*p* = 0.030), exocytosis with either neutrophils or eosinophils (*p* = 0.016), dermal infiltrates composed of lymphocytes, eosinophils, and neutrophils (*p* = 0.012), and absence of Langerhans cell microabscesses (*p* < 0.001) were more common in pemphigus than control. Spongiosis in pemphigus may mimic eczema in patients without acantholysis. The subtle histological findings in this study provide diagnostic clues and suggest that further immunofluorescence should be performed to confirm pemphigus diagnosis.

## 1. Introduction

Pemphigus is a group of chronic blistering disorders caused by autoantibodies that target various desmosomal proteins in the epidermis [1]. The classic lesions associated with pemphigus are flaccid blisters on the skin and mucosal surfaces. However, these blisters may not be appreciated because their fragility and transient nature can lead to various histological manifestations.

The diagnosis of pemphigus is based on clinical grounds, histopathology, and direct immunofluorescence (DIF) [2,3]. On histology, the most common hallmark pattern among all types of pemphigus is acantholysis, which is suprabasal in pemphigus vulgaris and subcorneal in pemphigus foliaceus. However, acantholysis may be present in other non-immunobullous diseases such as Grover’s disease, in arthropod bite reactions, and in herpes viral infection. Therefore, the presence of intercellular immunoglobulin (Ig) G and/or IgA assessed by DIF is usually required to confirm pemphigus diagnosis [4].

Acantholysis may be absent in biopsy of some cases of pemphigus. Instead, they may present with spongiosis and vesicle formation. The vesicle contents are usually composed of a few acantholytic cells, and occasionally neutrophils and eosinophils [5]. These non-specific findings may lead to a misdiagnosis of eczema [6].

Eosinophilic spongiosis and neutrophilic spongiosis have been described in pemphigus patients without acantholysis. These changes are rare [6,7,8,9,10]. Eosinophilic spongiosis has been noted in the periphery of acantholytic vesicles associated with dermal infiltrates composed of lymphocytes and eosinophils [6,7,9,10]. Neutrophilic spongiosis was commonly associated with IgA pemphigus, but reports have shown its presence in pemphigus vulgaris and pemphigus foliaceus as well [9,11,12]. Very few published studies have examined the exact nature of these histological changes. The aim of this retrospective study was to analyze the histopathologic features of spongiosis in DIF-confirmed cases of pemphigus, especially non-bullous lesions and periphery of blisters, in order to better understand the spongiosis occurring in pemphigus compared to acute eczematous dermatitis.

## 2. Materials and Methods

### 2.1. Study Design

This was a single-center, retrospective review of skin biopsy specimens. Skin biopsy slide specimens with pemphigus from 2001 to 2020 were retrieved from the dermatopathology database of our institution. Our institution is a tertiary referral hospital, accredited internationally for its dermatopathology program, and immunofluorescence diagnostic center. This study was performed with the approval of our Institutional Review Board (21MMHIS243e).

### 2.2. Inclusion and Exclusion Criteria

We retrieved all skin biopsy slide specimens with DIF records of immunopathological presence of intercellular IgG or IgA in the epidermis. Patients with pathologically diagnosed pemphigus without DIF confirmation, or with positive DIF without clinical correlation with pemphigus, were excluded. The hematoxylin and eosin (H&E)-stained slides of the included cases were further reviewed by two authors. Cases with spongiosis, either within the blisters or located in the peripheral epidermis, were selected for detailed pathological analysis.

For comparison purposes, we also retrieved all skin biopsy slide specimens of eczematous dermatitis or spongiotic dermatitis from 2020. Only patients with acute spongiotic dermatitis were included in the control group. Patients with subacute or chronic eczematous or inflammatory diseases with multiple histological patterns, such as psoriasiform spongiotic dermatitis, were excluded.

### 2.3. Histopathological Analysis

The following features were studied in H&E-stained slides. (1) The presence and level of vesicle formation; vesicles may develop subcorneally, intraepidermally, suprabasally, or be absent. (2) The presence of acantholysis. (3) The vesicle contents; these may contain acantholytic cells, neutrophils, eosinophils, or a mixture of these cells. (4) The presence and location of spongiosis in the blisters; spongiosis may develop in the upper, middle, or lower third of the epidermis. (5) The presence and location of spongiosis at the periphery of the blister; spongiosis may develop in the upper, middle, or lower third of the epidermis. (6) The type of cells infiltrating the spongiosis; predominantly, these may be eosinophils, neutrophils, or lymphocytes. (7) The intensity of dermal inflammation; this could be mild, moderate, or dense. (8) The pattern of inflammation; this could be classified as superficial perivascular, superficial and deep perivascular, interstitial, perifollicular, or mixed. (9) The type of dermal inflammatory infiltrate; this could include lymphocytes, histiocytes, eosinophils, neutrophils, or a mix of these cells. (10) The presence or absence of Langerhans cell, based on identification of microabscesses composed of more than three Langerhans cells.

### 2.4. Statistical Analysis

Descriptive statistics were employed in this study using Microsoft Excel 2021 for Windows. Fisher’s exact test was used to test the significance of the differences in categorical variables between two groups. One-way analysis of variance and post hoc *t*-tests were used for between-groups comparisons. Statistical significance was set at *p* < 0.05.

## 3. Results

### 3.1. Specimens Retrieved

A total of 99 immunopathological records of intercellular IgG or IgA deposition in the epidermis were identified within the study period. Two patients with morphea had false-positive result, presenting with weak intercellular IgG depositions, were excluded from the study. The total 97 confirmed cases of pemphigus included the following: 55 cases of pemphigus vulgaris, 32 pemphigus foliaceus, 3 pemphigus erythematosus, 2 paraneoplastic pemphigus, 2 pemphigus vegetans, 2 IgA pemphigus, and 1 pemphigus herpetiformis. Among them, 74 patients (76%) had pemphigus in the pathological diagnosis. Nineteen patients had nonspecific pathological diagnosis, such as ulceration, epidermal necrosis, perivascular dermatitis, or dermal fibrosis. Six patients were diagnosed as spongiotic dermatitis.

A total of 41 (42%) patients had obvious spongiosis in their skin biopsy specimens, either within the blisters or in their periphery. The enrolled cases included 24 cases of pemphigus vulgaris, 12 cases of pemphigus foliaceus, 2 subcorneal pustular dermatosis-type of IgA pemphigus, 1 pemphigus vegetans, 1 pemphigus erythematosus, and 1 pemphigus herpetiformis showing upper epidermal separation. Two major groups were used for statistical analyses: PV, referring to pemphigus vulgaris, represented cleavage at the suprabasal level or in the lower epidermis and included cases of pemphigus vulgaris and pemphigus vegetans (25 cases in total, 61%). SP, referring to superficial pemphigus, represented cleavage at the subcorneal level or in the upper epidermis and included pemphigus foliaceus, pemphigus erythematosus, IgA pemphigus, and pemphigus herpetiformis (16 cases in total, 39%). Fifteen patients were men and twenty-six were women, and the median patient age was 62 (29–85) years.

In the control group, 82 slide specimens with a diagnosis of eczematous dermatitis or spongiotic dermatitis from 2020 were identified. Of the 82 slide specimens, 20 were diagnosed as having acute spongiotic dermatitis. Ten of these patients were men and ten were women, with a median patient age of 46 (14–80).

### 3.2. Epidermal Changes in Cases of Pemphigus with Obvious Spongiosis

**Figure 1:**
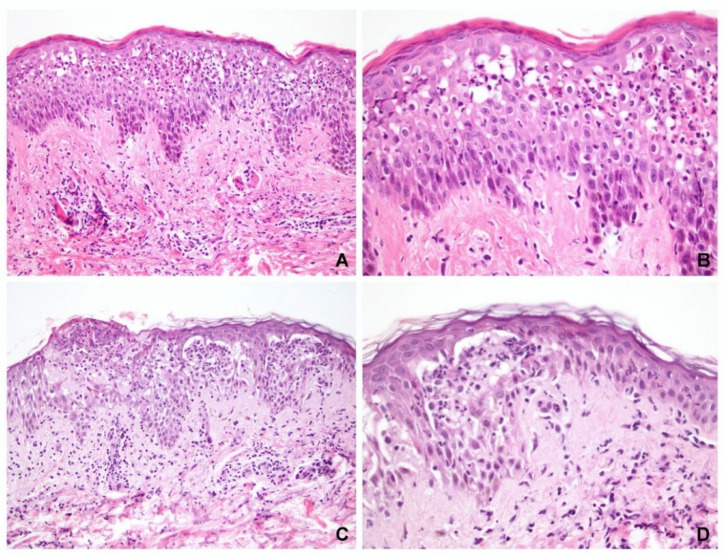
Spongiosis and inflammatory cell exocytosis in non-bullous pemphigus. (**A**,**B**) A case of pemphigus foliaceus showed diffuse spongiosis in the middle to upper epidermis with predominant neutrophil exocytosis in the middle epidermis. (**C**,**D**) A case of pemphigus vulgaris demonstrated diffuse spongiosis with microvesicle formation in the lower epidermis. Both cases did not have blisters in the biopsy specimens but could be diagnosed as pemphigus pathologically. The inflammatory cell exocytosis was located in the similar level of the epidermis. (Hematoxylin and eosin, original magnification, (**A**) 200×; (**B**) 400×; (**C**) 200×; (**D**) 400×).

Of the 41 specimens with obvious spongiosis, vesicle formation was observed in 33 and acantholysis was observed in 30 cases (73%). Eleven cases did not exhibit acantholysis (seven PV and four SP). Eight specimens (five PV and three PF) did not exhibit either blister formation or acantholysis, of which four cases exhibited epidermal ulcerations only (three PV and one SP), and in four other cases (two PV and two SP), pathological diagnosis of pemphigus can be made based on the presence of diffuse eosinophilic spongiosis or neutrophilic spongiosis (Figure 1).

**Figure 2:**
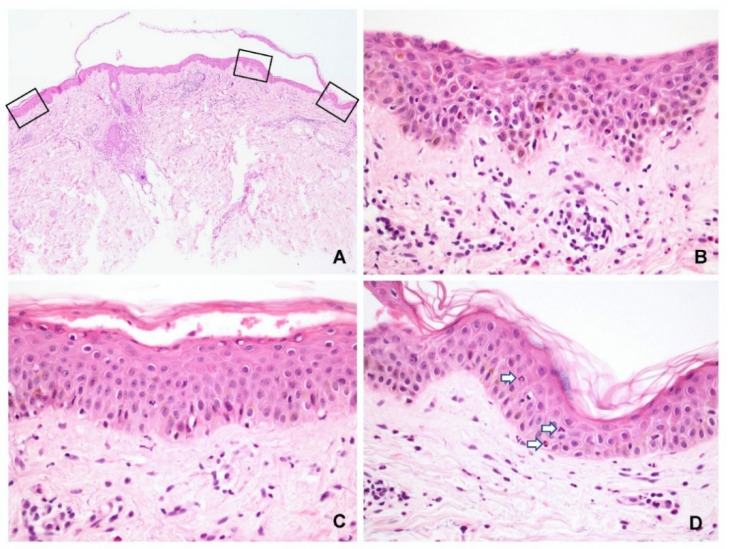
Spongiosis in pemphigus foliaceus. (**A**) Representative specimen showing a subcorneal blister. (**B**) Higher magnification of the center of the blister showed spongiosis in the entire epidermis. (**C**) A small subcorneal cleft was present in the left periphery, indicating an early change. (**D**) The right periphery epidermis showed subtle changes of spongiosis and neutrophil and eosinophil exocytosis (arrow) in the middle epidermis. (Hematoxylin and eosin, original magnification, (**A**) 20×; (**B**) 100×; (**C**) 400×; (**D**) 400×).

**Figure 3:**
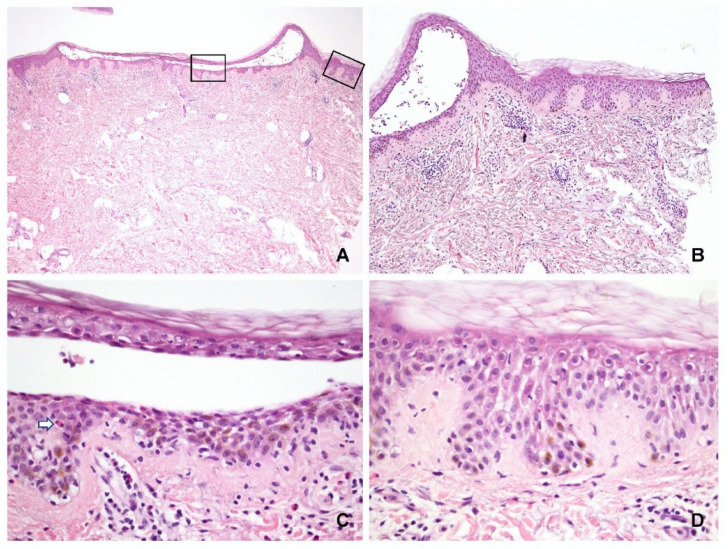
Spongiosis in pemphigus vulgaris. (**A**) Representative specimen showing an intraepidermal separation in the middle of the epidermis. (**B**) The periphery of the blister had diffuse spongiosis. (**C**) Higher magnification of the center of the blister showed diffuse spongiosis in both upper and lower epidermis. Lymphocyte and eosinophil exocytosis (arrow) could be seen. (**D**) Higher magnification of the periphery highlighted the spongiosis was present in the same level of the middle epidermis, that was different from spongiosis in the acute eczematous dermatitis. (Hematoxylin and eosin, original magnification, (**A**) 20×; (**B**) 100×; (**C**) 400×; (**D**) 400×).

The characteristic features of spongiosis in PV and SP are shown in Figure 2 and Figure 3, respectively. Spongiosis within the blisters was observed in the: lower third of the epidermis in 13 specimens (11 PV and 2 SP); lower to middle third in 15 specimens (10 PV and 5 SP); and entire epidermis in 10 specimens (1 PV and 9 SP). Three specimens (all PV) displayed no spongiosis within the blisters. These findings indicated that almost all SP cases had spongiosis involving not only the upper but also the lower epidermis (Figure 2A,B).

In the periphery of the lesion, spongiosis was observed (Figure 2D and Figure 3D) in the: lower third of the epidermis in 7 specimens (6 PV and 1 SP); lower to middle third in 14 specimens (11 PV and 3 SP); and entire epidermis in 5 specimens (1 PV and 4 SP). Fifteen specimens (seven PV and eight SP) displayed no spongiosis in the peripheral epidermis.

**Figure 4:**
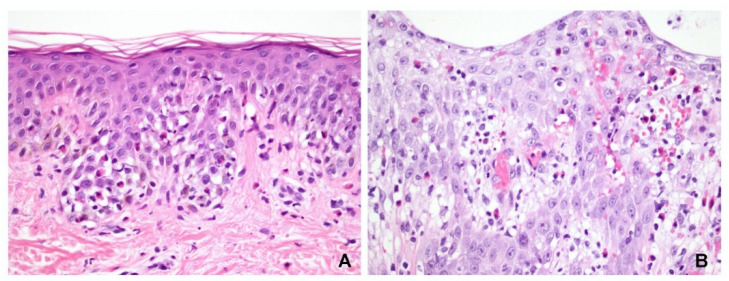
Eosinophilic spongiosis could be seen in either (**A**) pemphigus vulgaris or (**B**) acute eczematous dermatitis. The spongiosis and eosinophil exocytosis were more localized in the lower epidermis in pemphigus. The distribution of eosinophils in the exocytosis was more discrete in different levels of the epidermis in eczematous dermatitis. (Hematoxylin and eosin, original magnification, (**A**) 400×; (**B**) 400×).

Of the 41 slide specimens, 9 had only one type of inflammatory cell infiltration: 5 cases were associated with lymphocytic infiltration, 2 with neutrophilic, and 2 with eosinophilic. The remaining 32 specimens had mixed cell infiltration; specifically, lymphocyte-predominant infiltration was observed in 2 samples, neutrophil-predominant in 16, and eosinophil-predominant in 14. Of the 18 neutrophil-predominant exocytosis specimens, 9 were PV and 9 were SP. However, of the 16 eosinophil-predominant exocytosis specimens, 13 were PV and only 3 were SP. Therefore, whereas neutrophilic spongiosis is observed in both PV and SP, eosinophilic spongiosis appears to be more common in PV. However, there was no statistically significant difference between the two groups (*p* = 0.080, Fisher’s test) (Figure 4). Langerhans cell microabscess was not observed in any of the pemphigus specimens.

### 3.3. Dermal Changes in Cases of Pemphigus with Obvious Spongiosis

All 41 specimens had dermal inflammation involving the superficial plexus, 13 had an interstitial component, and 4 had perifollicular infiltration. The dermal infiltrates observed in all specimens were of the mixed type, composed of lymphocytes, neutrophils, and eosinophils. In terms of the intensity of dermal inflammation, 21 specimens were associated with mild, 14 specimens with moderate, and 6 specimens with dense inflammation.

### 3.4. Control Group

**Figure 5:**
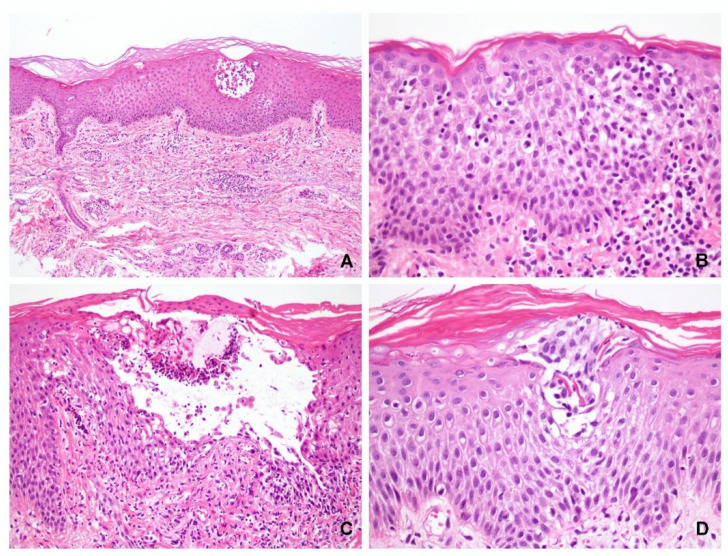
Spongiotic dermatitis. (**A**) The spongiosis was more commonly observed in the upper and middle epidermis. (**B**) Most inflammatory cells in the exocytosis were lymphocytes. (**C**) Acantholytic keratinocytes, some eosinophils and neutrophils could be present in the spongiotic microvesicle. (**D**) Langerhans cell microabscesses were present in eczematous dermatitis but usually absent in pemphigus. (Hematoxylin and eosin, original magnification, (**A**) 100×; (**B**) 400×; (**C**) 200×; (**D**) 400×).

Among cases reported in 2020, 82 slide specimens with a diagnosis of spongiotic dermatitis could be identified; of those, 20 fulfilled the inclusion criteria of this study. Intraepidermal vesicle formation was observed in 12 of these specimens, including 4 located in the upper epidermis and 8 located in both the upper and middle epidermis (Figure 5A,B). Interestingly, acantholysis was found in 11 (55%) of these specimens, but it did not separate at the same level as the epidermis (Figure 5C).

Within the lesion, most cases (15/20, 75%) had spongiosis involving the entire epidermis, three involved the lower epidermis only, and two involved the lower to middle epidermis. In other words, 15 cases had spongiosis in the upper third, 17 in the middle third, and 3 in the lower third of the epidermis. The exocytotic inflammatory cells in the spongiotic areas were lymphocytes in only 15 specimens, and lymphocytes with predominant eosinophils in 5 specimens. A small number of eosinophils or neutrophils could be seen within the microvesicles in 10 cases. Langerhans cell microabscesses were found in the upper third of the epidermis in 11 (55%) specimens (Figure 5D).

All specimens presented a superficial perivascular pattern, with eight specimens having both perivascular and interstitial patterns. Dermal infiltrates were composed of lymphocytes and histiocytes in all specimens, whereas eosinophils were also noted in 11 specimens. The intensity of dermal inflammation was dense in seven, moderate in eight, and mild in five specimens.

### 3.5. Comparison between Groups

There was no statistically significant difference in spongiosis levels within the blister. However, different levels of spongiosis were observed in the peripheral epidermis when comparing the pemphigus and control groups (*p* = 0.030). The pemphigus group (especially PV) tended to have spongiosis in the lower epidermis compared to the eczematous dermatitis group.

**Figure 6:**
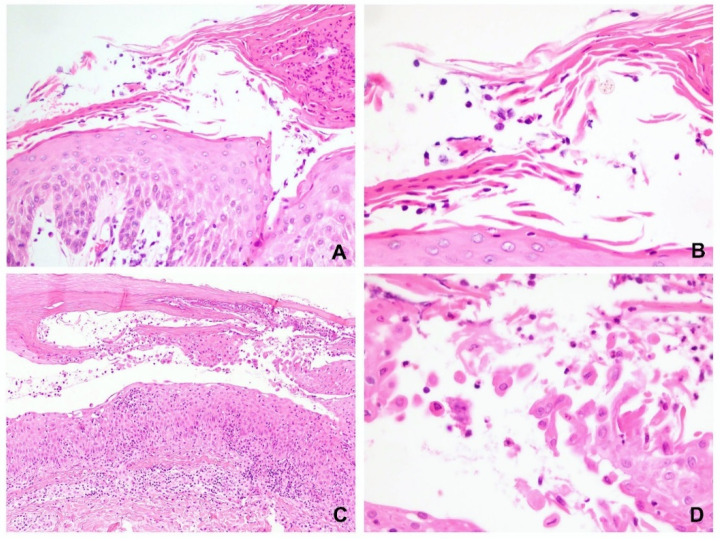
Acantholysis in the control group of spongiotic dermatitis. (**A**,**B**) The acantholysis might occur superficially in the stratum corneum and granular cell layer that mimicked superficial pemphigus. (**C**,**D**) Intraepidermal acantholytic keratinocytes could be prominent in spongiotic dermatitis. However, the acantholysis only located within the microvesicles and did not present in the periphery epidermis. (Hematoxylin and eosin, original magnification, (**A**) 100×; (**B**) 400×; (**C**) 100×; (**D**) 400×).

Acantholysis was more frequently present in the pemphigus group (72% in PV and 75% in SP) but was also frequently seen in the control group (55%). Therefore, the presence of acantholysis in the spongiotic area may not always indicate pemphigus (*p* = 0.358). Two cases in the control group had acantholysis occurred in the subcorneal area that mimicked SP (Figure 6A,B). Nine cases of spongiotic dermatitis had intraepidermal acantholysis (Figure 5C and Figure 6C,D). They were all acantholytic cells floating in the microvesicles. The acantholysis was not observed in the peripheral epidermis. Inflammatory cell exocytosis also differed between the pemphigus and control groups (*p* = 0.016). In addition, more leukocytes, either neutrophils or eosinophils, infiltrated the epidermis in the pemphigus group.

There were no statistically significant differences in the pattern and intensity of dermal inflammation between the groups. However, dermal infiltration in the pemphigus group had more leukocytes, including both neutrophils and eosinophils, compared to the control group which mostly had lymphocytic and occasionally eosinophilic infiltration (*p* = 0.012). No Langerhans cell microabscesses were found in the pemphigus group, thereby making the presence of Langerhans cell microabscesses a useful finding for distinguishing the two groups (*p* < 0.001). These results are summarized in Table 1.

## 4. Discussion

Spongiosis was present in approximately half of the retrieved specimens with a confirmed pemphigus diagnosis, including PV and SP, and presented in both the blistering and peripheral epidermis. Leukocyte exocytosis, either neutrophilic, eosinophilic, or both, was more often observed in pemphigus than in eczematous dermatitis, making it a useful biomarker for identifying the non-bullous stage of pemphigus. The presence of Langerhans cell microabscess, on the other hand, was more frequent in eczematous dermatitis. Acantholysis was observed in both pemphigus and eczematous dermatitis; however, the pattern was different.

About one-fifth of pemphigus cases in this study did not show the characteristic finding of acantholysis and displayed only ulceration or peripheral spongiosis in the epidermis. Crotty et al. and Manocha et al. [13] reported similar findings in pemphigus slides presenting with neutrophilic or eosinophilic spongiosis, or subtle intraepidermal microvesicles. Kouskoukis and Ackermann [14] reported the presence of vacuole-forming vesicles in the upper part of the epidermis. Compared with control spongiotic dermatitis specimens, some pemphigus cases in our study may display similar vesicle content and location, and DIF should be performed to distinguish the two conditions.

The spongiosis found within the blistering or peripheral epidermis was consistently located in the lower third of the epidermis in either the PV or SP, with some cases reaching the middle third of the epidermis. Spongiosis in the upper third was mostly SP, but this was also the case in the control group. Therefore, inflammatory exocytosis is important for differentiating between pemphigus and eczema. Neutrophils and eosinophils predominantly infiltrated the spongiotic areas in the pemphigus group. Previous studies have suggested that although neutrophilic and eosinophilic spongiosis are rare, they have been documented in pemphigus [6,8,9,10,11,12]. Our findings suggested that the presence of spongiosis predominantly infiltrated with neutrophils or eosinophils in the lower to middle thirds of the epidermis may be a helpful clue in cases of pemphigus without prominent acantholysis (Figure 1 and Figure 4).

Acantholysis, the hallmark for the diagnosis of pemphigus, was interestingly commonly found in the control group of spongiotic dermatitis. Fortunately, these were all floating acantholytic keratinocytes in the microvesicles accompanied by the presence of a small number of eosinophils and/or neutrophils. The pattern was different from diffuse acantholysis in pemphigus. The acantholysis might be attributed to spongiosis-related keratinocyte dyscohesion or lytic enzyme released from exocytotic leukocytes.

All specimens of pemphigus had superficial perivascular and interstitial infiltrates mostly composed of lymphocytes, neutrophils, and eosinophils. The control group displayed the same pattern of inflammation; however, it was mostly infiltrated by lymphocytes, histiocytes, and eosinophils. Although the intensity of inflammation between the two groups was not significant, the authors noted that the intensity of dermal inflammation in the control group coincided with vesicle formation in contrast to the pemphigus group, where vesicle formation occurred despite the intensity of dermal inflammation.

The presence of Langerhans cell microabscesses supports the pathological diagnosis of contact dermatitis [15]. In our study, Langerhans cell microabscesses were seen in more than half of the control group, whereas they were absent in the entire pemphigus group. Studies on the role of Langerhans cells in the pathogenesis of pemphigus are limited. In a report by Blitstein-Willinger [16], Langerhans cells were minimally detected in pemphigus compared to normal skin. Moreover, Santi et al. [17] found that Langerhans cells were decreased in lesional skin compared with perilesional skin in pemphigus. These results may explain the absence of Langerhans cell microabscesses in pemphigus cases reported in the present study. Langerhans cells have been suggested to suppress autoimmunity by suppressing regulatory T cells. The loss of Langerhans cells in skin lesions has been attributed to loss of suppression of these regulatory T cells [18]. Further studies are warranted to elucidate the role of these cells in the development of pemphigus.

The limitations of our study include the relatively small sample size, and sample retrieval from a single institution. Moreover, immunohistochemical staining was not performed to specifically identify the distribution of Langerhans cells.

In conclusion, our study suggests that the presence of spongiosis, predominantly infiltrated by neutrophils or eosinophils, in the lower to middle thirds of the epidermis, as well as the absence of Langerhans cell microabscesses may provide subtle clues indicative of pemphigus. Additional diagnostic tests such as immunofluorescence should be performed to confirm the diagnosis of pemphigus.

## Figures and Tables

**Table 1:** Histopathological comparison between the pemphigus and control groups.

Pathological Findings	PV * (*N* = 25)	SP * (*N* = 16)	All * (*N* = 41)	Control (*N* = 20)	*p* Value between Groups
*Location of spongiosis (within the blister)*					
Upper third epidermis	1	9	10	12	
Middle third epidermis	11	14	25	8	
Lower third epidermis	22	16	38	0	
None	3	0	3	0	
*p* value (versus control)	0.139	0.128	0.099	-	0.377
*Location of spongiosis (periphery)*					
Upper-third epidermis	1	4	5	12	
Middle-third epidermis	12	7	19	8	
Lower-third epidermis	18	8	26	0	
None	7	8	15	0	
*p* value (versus control)	0.076	0.119	0.030	-	0.077
*Presence of blister*					
Presence	20	13	33	12	
*p* value (versus control)	0.933	0.946	0.955	-	0.232
*Level of blister*					
Subcorneal	0	13	13	3	
Intraepidermal	14	0	14	9	
Suprabasal	6	0	6	0	
None	5	3	8	8	
*p* value (versus control)	0.448	0.215	0.187		0.171
*Presence of acantholysis*					
Presence	18	12	25	11	
*p* value (versus control)	0.358	0.348	0.301	-	0.358
*Type of cell infiltrate in spongiotic area*					
Lymphocyte-predominant	3	4	7	15	
Neutrophil-predominant	9	9	18	0	
Eosinophil-predominant	13	3	16	5	
*p* value (versus control)	0.059	0.232	0.016	-	0.203
*Patterns of dermal infiltration*					
Perivascular	18	10	28	12	
Perivascular and interstitial	7	6	13	8	
Perifollicular	4	0	4	0	
*p* value (versus control)	0.380	0.430	0.267	-	0.848
*Intensity of dermal inflammation*					
Mild	13	8	21	5	
Moderate	8	6	14	8	
Intense	4	2	6	7	
*p* value (versus control)	0.271	0.116	0.299	-	0.433
*Type of cell infiltrate in the dermis*					
Lymphocytes, neutrophils, eosinophils	24	15	39	0	
Lymphocytes, eosinophils	1	1	2	11	
Lymphocytes	0	0	0	9	
*p* value (versus control)	0.007	0.007	0.012	-	0.003
*Langerhans cell microabscess*					
Presence	0	0	0	11	
*p* value (versus control)	<0.001	<0.001	<0.001	-	<0.001

* PV: pemphigus vulgaris group with lower epidermal separation; SP: superficial pemphigus group with upper epidermal separation; All: all pemphigus specimens with spongiosis.

## Data Availability

Data are contained within the article.
